# Age at First Pregnancy, Adult Weight Gain and Postmenopausal Breast Cancer Risk: The PROCAS Study (United Kingdom)

**DOI:** 10.1002/ijc.70489

**Published:** 2026-04-12

**Authors:** Lee Malcomson, Adam Brentnall, Andrew G. Renehan, Mary Pegington, Elaine F. Harkness, Jake Southworth, D. Gareth Evans, Michelle Harvie

**Affiliations:** ^1^ Division of Cancer Sciences, Faculty of Biology, Medicine and Health University of Manchester Manchester UK; ^2^ Wolfson Institute of Population Health Queen Mary University of London London UK; ^3^ Nightingale Breast Screening Centre & the Prevent Breast Cancer Research Unit Manchester University NHS FT Manchester UK; ^4^ Division of Informatics, Imaging and Data Sciences, Faculty of Biology, Medicine and Health University of Manchester Manchester UK; ^5^ Division of Evolution, Infection and Genomics, Faculty of Biology Medicine and Health University of Manchester Manchester UK; ^6^ Manchester Academic Health Science Centre Manchester UK

**Keywords:** age at first pregnancy, breast cancer, parity, weight gain

## Abstract

Adult weight gain (AWG) increases postmenopausal breast cancer risk, whereas an early first pregnancy (FP) is protective. As pregnancy is a key contributor to weight gain, we investigated a potential interaction effect between these two factors on BC risk. We analysed prospective data from 48,417 women in the Predicting Risk of Breast Cancer at Screening (PROCAS) cohort (recruited 2009–2015). A Cox proportional hazards model was used to test for an interaction between first pregnancy age and weight gain on breast cancer risk. After a median follow‐up of 6.4 years, 1702 incident breast cancers were identified. Compared to women with an early FP (< 30 years) and stable weight (≤ 5%), risk was highest among those with substantial AWG (> 30%) combined with a late FP (≥ 30 years) (HR: 2.48, 95% CI: 1.82–3.37) or nulliparity (HR: 2.38, 95% CI: 1.74–3.27). Elevated risk was observed even with moderate weight gain (5%–15%). A non‐significant positive trend toward an additive interaction was observed for late FP (Relative Excess Risk due to Interaction (RERI): 0.32), whereas the risk in nulliparous women appeared independent of weight gain (RERI: −0.05). Maintaining a stable adult weight and an early first pregnancy are independently associated with a lower breast cancer risk. However, adult weight gain remains a significant risk factor regardless of reproductive history. The combination of high weight gain and late or no pregnancy identifies a high‐risk group who could be prioritized for weight‐management interventions in cancer prevention settings.

## Abstract (highlights)

What's new?

An early first pregnancy is protective against postmenopausal breast cancer. However, pregnancy is also a key contributor to weight gain, and adult weight gain increases postmenopausal breast cancer risk. This prospective cohort study quantified the combined effect of adult weight gain and age at first pregnancy on postmenopausal breast cancer risk, showing that the protective effect of an early first pregnancy does not attenuate the risk associated with weight gain. Moreover, the study identifies women with late or no pregnancy who gain substantial weight (> 30%) as a high‐risk group who could benefit from prioritized weight‐management interventions in cancer prevention settings.

## Graphical abstract

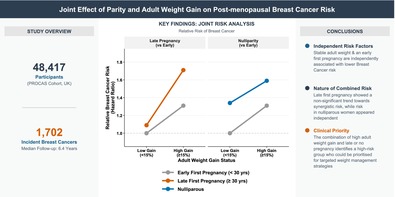

**Abbreviations**
ANOVA: analysis of variance
AP: attributable proportion
AWG: adult weight gain
BC: breast cancer
BMI: body mass index
CCA: complete case analysis
CI: confidence interval
DAG: directed acyclic graph
DCIS: ductal carcinoma in situ
ER + ve: oestrogen receptor positive
ER−ve: oestrogen receptor negative
FP: first pregnancy
HR: hazard ratio
HRT: hormone replacement therapy
HSD: honest significance difference
IMD: index of multiple deprivation
KM: Kaplan–Meier
NBSS: national breast screening system
NWCIS: north‐west cancer intelligence service
PROCAS: predicting risk of breast cancer at screening
RERI: relative excess risk due to interaction
SD: standard deviation
SES: socioeconomic status
SI: synergy index

## 1 Background

Breast cancer (BC) is the most frequently diagnosed cancer in the UK, with over 56,000 new cases between 2017 and 2019 [1]. Two established risk factors for postmenopausal BC are adult weight gain (AWG) [2] and a first pregnancy (FP) at a relatively late age (≥ 30 years) or nulliparity [3]. Understanding how these two factors interact to affect BC risk is important, as pregnancy itself is a key driver of long‐term weight gain in women [4]. In the UK, 35% of women retain over 5 kg 6 months postpartum [5], while in the United States, nearly half (47.7%) retain at least 4.5 kg 1 year postpartum [6]. Both AWG and the age of first pregnancy in the United Kingdom are increasing, with the proportion of the women classed as obese or overweight increasing from 49% in 1993 to 59% in 2021 [7] and the proportion of women giving birth over the age of 30 increasing from 30.9% in 1993 to 58.2% in 2021 [8].

Given these trends and with U.K. female BC diagnoses at their highest recorded rate [1], there is a need to investigate how these factors interact on BC risk to better inform and target weight control advice and interventions to those at greatest risk. This study aimed to determine whether the association between AWG and BC risk is modified by first pregnancy age, specifically whether an early first pregnancy attenuates the adverse effect of weight gain during adulthood.

## 2 Methods

### 2.1 Population

The Predicting Risk of Breast Cancer at Screening (PROCAS) cohort study, described in full elsewhere [9], recruited 57,902 women aged 46–84 years from the Greater Manchester Breast Screening Program between October 2009 and June 2015. Each consented participant completed a single two‐page questionnaire at the time of recruitment. This questionnaire captured self‐reported information about BC risk factors, current weight, and recalled weight at age 20.

**FIGURE 1:**
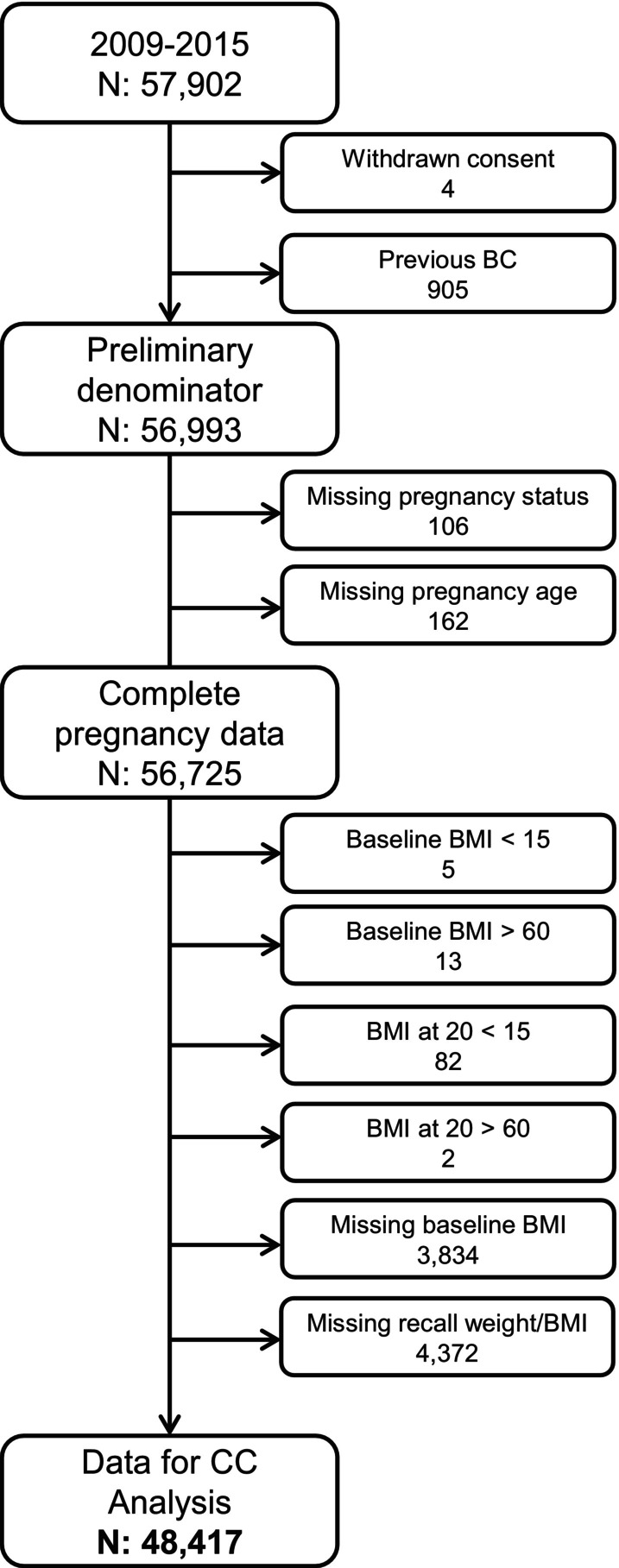
Study flow diagram. This flow diagram illustrates the selection process for the final study cohort. It begins with the initial 57,902 participants recruited for the PROCAS study and details the number of individuals excluded at each stage, resulting in the final 48,417 participants used for the Complete Case Analysis.

We undertook a complete case analysis (CCA), including 48,417 participants (Figure 1) who provided information on pregnancy status and AWG. From the total cohort of 57,902 participants, the following were excluded: Four withdrew consent for their data to be used for research purposes; 905 due to a previous BC diagnosis at the time of recruitment; 268 participants with missing data for pregnancy status or pregnancy age; 102 participants with outlier BMI recordings at either time point (defined as BMI < 15 or > 60—due to the possibility of these being recording errors); 3834 participants with a missing baseline BMI; and 4372 participants missing recall weight at 20.

### 2.2 Exposure Measurements

Relative adult weight change (%) was determined from self‐reported weights recalled by the participant at age 20 (representing early adulthood), and their self‐reported weight at study entry (representing mid‐to‐late life, median age at study entry: 57). This measure therefore reflects the estimated cumulative AWG over this period.

Women were categorized as having either an early FP < 30 years, a late FP ≥ 30 years, or as nulliparous. This was derived from the self‐reported question: “How old were you at your first pregnancy?”. We acknowledge that participants likely interpreted this as age at first birth rather than conception, although interpretation may have varied. The use of age 30 as a key threshold is supported by a meta‐analysis of over 100 studies, which demonstrated a marked increase in BC risk for women having a first pregnancy at age 30 or older [10].

### 2.3 Outcome Measures

The primary outcome was the diagnosis of a new BC, classed as either an invasive or ductal carcinoma in situ (DCIS), diagnosed after the study entry date. Only the first BC diagnosis was considered for each participant, with any secondary or recurrent BCs excluded from the analysis.

The study followed participants from their PROCAS enrolment date (between October 2009 and February 2013) to a final data query for any cancer diagnoses performed in January 2022. Cancer events were identified through the Somerset Cancer Registry and the North‐West Cancer Intelligence Service (NWCIS). For event‐free participants, the date of their most recent mammogram, obtained from the National Breast Screening System (NBSS), was used as the censoring date for follow‐up. This censoring date was selected to confirm active residency within the catchment areas of the specific cancer registries used (NWCIS/Somerset), to prevent ascertainment bias from participants who may have moved out of the region. Therefore, while the covariate and exposure data were collected cross‐sectionally at baseline, cancer outcomes were ascertained prospectively through registry linkage.

### 2.4 Statistical Analysis

Differences between baseline characteristics were assessed by chi‐squared tests for categorical variables and one‐way analysis of variance (ANOVA) tests for continuous variables. Post hoc Tukey's Honest Significance Difference (HSD) tests were performed on variables of interest which returned a significant ANOVA result to quantify differences between groups.

**FIGURE 2:**
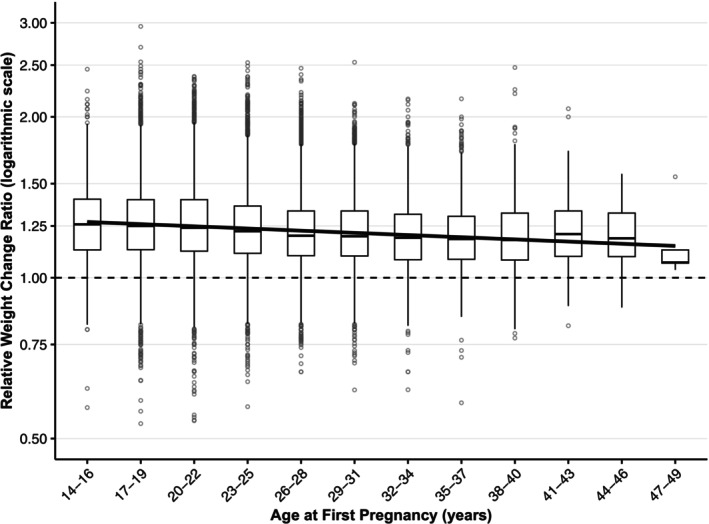
Box plot of relative weight change by age at first pregnancy. This plot shows the relationship between the age at first pregnancy and the relative adult weight gain. The *y*‐axis is on a natural logarithmic scale to accurately represent the variation between weight gain and loss across different first pregnancy age groups. Multiple linear regression adjusted for recruitment age and number of children indicated a significant inverse association. For every additional year of age at first pregnancy, relative adult weight gain decreased by *β* = −0.39% (95% CI: −0.43 to −0.35, *p* < 0.001), and absolute weight gain decreased by *β* = −0.21 kg (95% CI: −0.23 to −0.18, *p* < 0.001).

To accurately visualize the relationship between age of FP and AWG, a box plot with a natural logarithmic scale was created from a data subset excluding those who were nulliparous (Figure 2).

Multiple linear regression models were used to quantify the association between age at FP and AWG. The unstandardized beta coefficient (β) from these models represents the average change in weight gain (in % or kg) for each one‐year increase in age at FP. The covariates included within this model were age at recruitment to the PROCAS study and number of children. Age was included due to its anticipated correlation with BMI and number of children allowed for the analysis to be adjusted for those who had multiple full‐term pregnancies as it is the age of first pregnancy that is of interest.

To investigate the interaction between AWG and age at FP, we combined these risk factors into the following categorical groups:

- Group 1 (Referent): Early FP (< 30 years) with low AWG (< 15%)
- Group 2: Early FP with high AWG (≥ 15%)
- Group 3: Late FP (≥ 30 years) with low AWG
- Group 4: Late FP with high AWG
- Group 5: Nulliparous with low AWG
- Group 6: Nulliparous with high AWG

Kaplan–Meier (KM) failure estimates were plotted by parity, AWG and these defined factors, with BC diagnosis defined as the event. Participants without a BC diagnosis were censored at the date of their last follow up mammogram or date of death, whichever was later.

Covariates for the fully adjusted model were selected based on a Directed Acyclic Graph (DAG) constructed using knowledge of causal pathways between reproductive history, lifestyle, and BC risk (Figure S1). The final multivariable models were adjusted for the following adjustment set where data was available: recruitment age, BMI at age 20, HRT use, menopausal status, height, family history, ethnicity, age at menarche, alcohol consumption, physical activity, and the Index of Multiple Deprivation (IMD) 2010 Rank (used as a proxy for socioeconomic status (SES)).

The number of pregnancies was identified as a mediator on the causal pathway between age at first pregnancy and AWG and was excluded to prevent over‐adjustment bias. The interaction of BMI at age 20 and HRT use was also included based on evidence that the effect of HRT on BC risk has been found to vary at different BMI levels as both high BMI and HRT use independently increase oestradiol levels, a hormone linked to a higher risk of BC [11]. Therefore, HRT has a greater impact on oestradiol levels and BC risk in someone with a lower BMI. To assess the influence of the full covariate set on the risk estimates, a sensitivity analysis was performed adjusting for recruitment age alone.

For the primary analysis, Cox Proportional Hazard modeling was used to estimate mean hazard ratios (HR) with 95% confidence intervals. AWG was first split into above and below 15% to allow comparison with the KM plots. This threshold was selected to define substantial weight gain while ensuring sufficient participant numbers in the combined risk groups to robustly test for statistical interaction. Additionally, to investigate the dose–response relationship, AWG was stratified into four categories: stable/loss (≤ 5%), moderate gain (> 5%–15%), high gain (> 15%–30%), and very high gain (> 30%). Finally, AWG was analyzed as a continuous measure to avoid any data loss from the use of categorical variables.

A potential interaction between high AWG (> 15%) and a late FP or nulliparous status on BC risk was assessed on both multiplicative and additive scales. Multiplicative interaction was evaluated by including a product term for the two exposures in the Cox regression model. To assess for additive interaction, we calculated the relative excess risk due to interaction (RERI), the Attributable Proportion due to Interaction (AP), and the Synergy Index (SI). As this method requires the use of binary variables, an interaction term of these factors was also included in the model with AWG used as a continuous measure. A likelihood ratio test was performed to determine whether including the interaction term resulted in an improved model.

Data cleaning, wrangling and analysis were carried out using RStudio 2021.09.1, with R version 4.2.1. The STROBE guidelines for reporting cohort studies [12] were followed to ensure consistent reporting, and the completed STROBE checklist is available in the Supporting Information.

## 3 Results

### 3.1 Participant Characteristics & Breast Cancer Risk Factors by Parity Status

The baseline characteristics are shown in Table 1 by parity status for the 48,417 participants included in the analysis. Almost three‐quarters of participants had an early FP (age < 30 years) (73.5%); the remainder were almost equally classed as late FP (age ≥ 30 years) (13.9%) and nulliparous (12.6%). The mean (sd) age of FP was 22.9 (3.5) in the early group and 33.2 (3.1) in the late group. Compared with women who had a late FP or were nulliparous, those with an early FP were older (median age 58.5 vs. 53.9 years) and had a higher median BMI at study entry (26.6 vs. 25.4 kg/m^2^). However, median BMI at age 20 was similar across all groups (21.3 vs. 21.6 kg/m^2^), indicating that the difference in study‐entry BMI is largely attributable to subsequent AWG.

**TABLE 1:** Baseline characteristics: 48,417 women in PROCAS (2009–2015) Dataset used for complete case analysis.

	Early first pregnancy < 30 years	Late first pregnancy ≥ 30 years	Nulliparous	Total
Number of women (% of total)	35,591 (73.5)	6733 (13.9)	6093 (12.6)	48,417
Ethnicity (% of total)				
White	32,944 (92.6)	6276 (93.2)	5680 (93.2)	44,900 (92.7)
Asian	450 (1.3)	104 (1.5)	52 (0.8)	606 (1.3)
Black	258 (0.7)	40 (0.6)	52 (0.8)	350 (0.7)
Jewish	314 (0.9)	86 (1.3)	62 (1.0)	462 (1.0)
Mixed	173 (0.5)	28 (0.4)	38 (0.6)	239 (0.5)
Other	275 (0.8)	73 (1.1)	75 (1.2)	423 (0.9)
Missing	1177 (3.3)	126 (1.9)	134 (2.2)	1437 (3.0)
At study entry				
Median age (IQR)	58.46 (52.3–64.4)	53.86 (50.7–59.9)	55.03 (51.0–61.4)	57.26 (51.8–63.7)
Median BMI (IQR)	26.57 (23.8–30.4)	25.42 (22.8–29.1)	25.61 (22.8–29.8)	26.34 (23.5–30.1)
Median height (m) (IQR)	1.60 (1.6–1.7)	1.63 (1.6–1.7)	1.63 (1.6–1.7)	1.63 (1.6–1.7)
Mean height (m) (SD)	1.61 (0.1)	1.63 (0.1)	1.63 (0.1)	1.62 (0.1)
Menopausal status				
Pre and Peri‐menopausal	10,329 (29.0)	2994 (44.4)	2331 (38.3)	15,654 (32.3)
Post‐menopausal	25,262 (71.0)	3739 (55.5)	3762 (61.7)	32,763 (67.7)
Median age at menopause (IQR)	50 (45–51)	50 (47–52)	49 (45–51)	50 (46–51)
Mean age at menopause (SD)^a^	48.03 (5.6)	48.9 (4.3)	47.82 (5.3)	48.11 (5.4)
HRT Type				
Oestrogen only (ever)	6369 (17.9)	522 (7.8)	745 (12.2)	7636 (15.8)
Combined (ever)	7723 (21.7)	1223 (18.2)	1218 (20.0)	10,164 (21.0)
Never	21,173 (59.5)	4934 (73.2)	4078 (66.9)	30,185 (62.3)
Unknown	326 (0.9)	54 (0.8)	52 (0.8)	432 (0.9)
Hysterectomy				
No	25,728 (72.3)	5859 (87.0)	4993 (82.0)	36,580 (75.6)
Yes	9629 (27.1)	829 (12.3)	1060 (17.4)	11,517 (23.8)
Unknown	234 (0.7)	46 (0.7)	40 (0.7)	320 (0.7)
Both ovaries removed	3205 (9.0)	364 (5.4)	534 (8.8)	4103 (8.5)
Alcohol use				
No	9794 (27.5)	1323 (19.6)	1378 (22.6)	12,495 (25.8)
Yes	25,284 (71.0)	5348 (79.4)	4654 (76.4)	35,286 (72.9)
Missing	513 (1.4)	62 (0.9)	61 (1.0)	636 (1.3)
If Yes, mean units per wk. (SD)	6.17 (7.9)	7.24 (7.9)	7.76 (8.8)	6.52 (8.1)
Mean age at menarche (SD)	12.87 (1.6)	12.93 (1.6)	12.85 (1.7)	12.88 (1.6)
Mean age 1st pregnancy (SD)	22.87 (3.5)	33.18 (3.1)	—	21.42 (9.4)
Any exercise past week:				
No	6437 (18.1)	1091 (16.2)	960 (15.8)	8488 (17.5)
Yes	25,750 (72.3)	5216 (77.5)	4723 (77.5)	35,689 (73.7)
Missing	3404 (9.6)	426 (6.3)	410 (6.7)	4240 (8.8)
If yes, mean hours past week (SD)	6.90 (9.5)	5.32 (7.6)	5.82 (7.8)	6.54 (9.1)
Family history of breast and/or ovarian cancer	10,075 (28.3)	1907 (28.3)	1694 (27.8)	13,676 (28.3)
Mean no. of children (SD)	2.44 (1.0)	1.76 (0.7)	—	2.04 (1.2)
Median IMD Rank^b^ (IQR)	14,353 (6019–23,133)	19,799 (11,625–26,925)	15,568 (7700–23,618)	15,289 (6969–23,915)
Median BMI at age 20 (IQR)	21.63 (20.0–23.4)	21.30 (19.8–23.1)	21.63 (19.1–23.6)	21.6 (20.0–23.4)
Median VAS Density (IQR)	23.13 (13.0–35.9)	28.75 (17.0–42.4)	30.25 (16.6–45.4)	24.63 (13.8–38.0)
Median follow‐up time (IQR) years	6.4 (3.5–9.1)	6.7 (5.8–9.2)	6.4 (5.1–9.1)	6.4 (4.4–9.1)

*Note:* ()—% of total, unless otherwise specified. The demographic, reproductive, and lifestyle characteristics of the 48,417 women included in the analysis, categorized by their parity status: early first pregnancy (< 30 years), late first pregnancy (≥ 30 years), or nulliparous.

^a^ For 25,262 participants recruited post‐menopause.

^b^ Lower IMD Rank = more deprived area.

Significant lifestyle and reproductive differences were also observed: women with an early FP were less likely to be current alcohol users (71.0% vs. 79.4% in late FP) and reported lower levels of recent physical activity (72.3% vs. 77.5%). Conversely, the early FP group had higher rates of historical hormonal exposure, including higher rates of hysterectomy (27.1% vs. 12.3%) and a different HRT profile, with fewer ‘never users’ (59.5% vs. 73.2%) compared to the late FP group.

### 3.2 The Relationship Between Age of First Pregnancy and Adult Weight Gain

Amongst parous participants, after adjusting for recruitment age and number of children, women with an early first pregnancy experienced greater AWG than those with a late FP (Figure 2). For every additional year of first pregnancy age, the estimated AWG decreased on average by 0.39% (95% CI: −0.43 to −0.35, *p* < 0.001) or 0.21 kg (95% CI: −0.23 to −0.18, *p* < 0.001).

### 3.3 Associations of Parity and Adult Weight Change With Breast Cancer Risk

**FIGURE 3:**
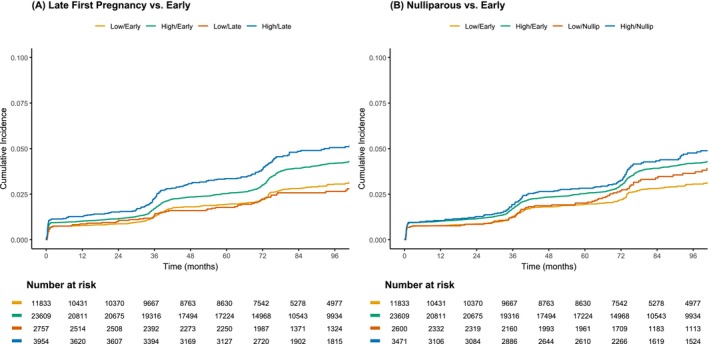
Kaplan–Meier failure estimates for breast cancer diagnosis by parity and adult weight change. Cumulative incidence of breast cancer by adult weight gain and parity status. (A) In women with a late first pregnancy (≥ 30 years), high weight gain (≥ 15%) is associated with a disproportionate increase in risk (synergy). (B) In nulliparous women, the risk increase appears additive, with a higher baseline risk observed even at low weight gain (< 15%).

After a median follow‐up of 6.4 years, there were 1702 new BC diagnoses (1605 invasive carcinomas and 97 DCIS). The Kaplan–Meier cumulative incidence estimates (Figure 3) visualize the risk of developing BC over time, stratified by late first pregnancy (Panel A) and nulliparity (Panel B) compared to early first pregnancy. Consistent across both panels, women with low AWG (< 15%) demonstrated the lowest cumulative incidence, suggesting a protective benefit regardless of reproductive history. Conversely, the highest risks were observed among those with high AWG (≥ 15%) combined with either a late first pregnancy or nulliparity. While cumulative incidence appeared higher for these subgroups compared to women with high AWG and an early first pregnancy, the differences between reproductive histories within the high‐weight category were not statistically significant.

KM failure estimates were also plotted by parity status alone (Figure S2), which showed similar levels of risk, although those who had an early FP showed a slight protection against the development of BC, particularly from 36 months after recruitment. This independent main effect was formally confirmed in our fully adjusted continuous models (Table S1), which demonstrated that for every one‐year increase in age at first pregnancy, BC risk independently increased (HR: 1.02, 95% CI: 1.00–1.04, *p* = 0.04). KM failure estimates were also plotted by AWG alone (Figure S3), which showed an increase in risk as AWG categories increased. When analyzed as a categorical variable, a clear dose–response relationship was evident. Furthermore, our fully adjusted continuous models (Table S1) confirmed this association, demonstrating that every 10% increase in relative AWG significantly increased the risk of BC (HR: 1.07, 95% CI: 1.04–1.10, *p* < 0.001).

### 3.4 Interactions of First Pregnancy Age and Adult Weight Gain on Breast Cancer Risk

A Cox proportional hazard model was estimated using AWG and parity as categorical variables (as above), testing for both multiplicative and additive interactions (Table 2). For women with a late FP, those with low AWG had a risk comparable to an early FP (HR: 1.07, 95% CI: 0.83–1.39), whereas those with high AWG had a significantly increased risk of 71% (HR: 1.71, 95% CI: 1.41–2.08). On the additive scale, this group showed a non‐significant positive trend toward an interaction effect with a RERI of 0.32 (95% CI: −0.07–0.70) and an attributable proportion (AP) of 0.18 (95% CI: −0.04–0.38). The interaction on the multiplicative scale was also positive but not statistically significant (HR: 1.23, 95% CI: 0.90–1.65).

**TABLE 2:** Interaction test of first pregnancy age and adult weight gain on breast cancer risk.

	Early first pregnancy (< 30 years)	Late first pregnancy (≥ 30 years)	Nulliparous	Late first pregnancy (≥ 30 years) or nulliparous
	HR [95% CI]	HR [95% CI]	HR [95% CI]	HR [95% CI]
Low adult weight gain (< 15%)	1 [Reference]	1.07 [0.83–1.39]	1.33 [1.04–1.71]	1.18 [0.97–1.46]
High adult weight gain (> = 15%)	1.32 [1.15–1.51]	1.71 [1.41–2.08]	1.60 [1.30–1.97]	1.66 [1.41–1.96]
Multiplicative scale	—	1.23 [0.90–1.65]	0.93 [0.67–1.24]	1.07 [0.83–1.34]
RERI	—	0.32 [−0.07–0.70]	‐0.05 [−0.50–0.36]	0.15 [−0.16–0.45]
AP	—	0.18 [−0.04–0.38]	‐0.04 [−0.34–0.21]	0.09 [−0.10–0.26]
SI	—	1.82 [0.80–8.14]	0.92 [0.47–2.10]	1.32 [0.78–2.97]

*Note:* This table details the results from the formal interaction analysis, showing the hazard ratios for breast cancer on both multiplicative and additive scales to assess the combined effect of high adult weight gain (≥ 15%) and a late (≥ 30 years) first pregnancy and nulliparous status. Adjusted for recruitment age + HRT use * BMI at 20 + menopausal status + alcohol + exercise + menarche age + ethnicity + height + family history + index of Multiple Deprivation. Interaction measures were calculated using the hazard ratios from the table: HR11 (High AWG & Late FP), HR10 (High AWG & Early FP), and HR01 (Low AWG & Late FP). Multiplicative Interaction: HR11/(HR10 × HR01); RERI (Relative Excess Risk due to Interaction): HR11—HR10—HR01 + 1; AP (Attributable Proportion): RERI/HR11; SI (Synergy Index): (HR11–1) / ((HR10–1) + (HR01–1)), with 95% CIs derived via Monte Carlo simulation. Model Fit: A Likelihood Ratio Test (LRT) comparing the model with and without the multiplicative interaction term indicated no statistically significant improvement in model fit (*p* = 0.69).

In contrast, nulliparous women exhibited an elevated risk even with low AWG (HR: 1.33, 95% CI: 1.04–1.71), which increased to 60% with high AWG (HR: 1.60, 95% CI: 1.30–1.97). Unlike the late pregnancy group, there was no evidence of synergistic interaction on the additive scale (RERI: −0.05, 95% CI: −0.50–0.36; AP: –0.04, 95% CI: −0.34–0.21) or the multiplicative scale (HR: 0.93, 95% CI: 0.67–1.24), suggesting the risk in nulliparous women acts independently of weight gain.

To better illustrate the relationship between AWG and parity, we analyzed AWG as a categorical variable (Table 3). We observed a clear dose–response relationship, where BC risk progressively increased with higher weight gain across all parity groups. Moderate weight gain (5% to < 15%) was associated with elevated risk, with HR ranging from 1.48 to 1.77 across the three groups compared to early first pregnancy women with stable weight. Risk further increased for those with high weight gain (15% to < 30%); specifically, women with a late first pregnancy in this category had a two‐fold increased risk (HR: 2.04, 95% CI: 1.49–2.79). The highest risks were observed amongst those with substantial AWG greater than 30%; in this category, women with a late first pregnancy had a significantly increased risk (HR: 2.48, 95% CI: 1.82–3.37), as did nulliparous women (HR: 2.38, 95% CI: 1.74–3.27).

**TABLE 3:** Hazard Ratios (HRs) for the association between categorical adult weight gain, first pregnancy age and breast cancer risk.

Adult weight change	Early first pregnancy (< 30 years)		Late first pregnancy (≥ 30 years)		Nulliparous	
	BC/*N* (%)	HR (95% CI)	BC/N (%)	HR (95% CI)	BC/*N* (%)	HR (95% CI)
Stable & Loss: ≤ 5%	81/3851 (2.10%)	1.00	27/1018 (2.65%)	1.28 [0.82–1.98]	34/1002 (3.39%)	1.68 [1.12–2.50]
Gain 5 to < 15%	201/6225 (3.23%)	1.48 [1.14–1.92]	45/1433 (3.14%)	1.48 [1.03–2.14]	45/1261 (3.57%)	1.77 [1.22–2.55]
Gain 15 to < 30%	325/9173 (3.54%)	1.60 [1.25–2.05]	79/1834 (4.31%)	2.04 [1.49–2.79]	58/1466 (3.96%)	1.81 [1.29–2.55]
Gain ≥ 30%	415/10485 (3.96%)	1.84 [1.44–2.34]	85/1651 (5.15%)	2.48 [1.82–3.37]	76/1542 (4.93%)	2.38 [1.74–3.27]

*Note:* Adjusted for recruitment age + HRT use * BMI at 20 + Menopausal status + alcohol + exercise + menarche age + ethnicity + height + family history + Index of Multiple Deprivation. This table displays the hazard ratios for developing breast cancer, stratified by four categories of adult weight gain and three categories of first pregnancy age (< 30 years vs. ≥ 30 years vs. nulliparous). The reference group consists of women with an early first pregnancy and stable weight or weight loss (≤ 5%).

To formally test for an interaction effect and prevent the data loss associated with using categorical variables, we repeated the analysis with weight change as a continuous variable (Table S1). In this analysis, age at first pregnancy (HR per year: 1.02, 95% CI: 1.00–1.04, *p* = 0.04) was independently associated with increased BC risk. However, there was no statistically significant multiplicative interaction effect between relative weight change and either late first pregnancy (HR: 1.01, 95% CI: 0.95–1.08, *p* = 0.68) or nulliparity (HR: 1.00, 95% CI: 0.94–1.07, *p* = 0.96). When the model was modified to replace relative weight change (%) with absolute weight change (kg), results remained consistent, showing no significant interaction for late first pregnancy (HR per kg: 1.00, 95% CI: 0.99–1.02, *p* = 0.57) or nulliparity (HR per kg: 1.00, 95% CI: 0.99–1.01, *p* = 0.87).

Comparison with a minimally adjusted model (controlling only for recruitment age) showed that additional adjustment for lifestyle and reproductive factors resulted in a slight attenuation of risk estimates (Late FP/High AWG: HR 1.81, 95% CI 1.52–2.17; Nulliparous/High AWG: HR 1.65, 95% CI 1.36–2.00) (Table S3). When modelling weight gain as a continuous variable within this model, the association with BC risk remained significant (HR per 10% increase: 1.07, 95% CI: 1.05–1.10), and consistent with the primary analysis, there was no evidence of a statistical interaction with parity (likelihood ratio test *p* = 0.77).

### 3.5 Sensitivity Analyses

In a sensitivity analysis stratifying by menopausal status at diagnosis (Table S2), associations in postmenopausal women (81.7% of cases) remained consistent with the primary findings (Late FP/High AWG: HR 1.68, 95% CI 1.33–2.11; Nulliparous/High AWG: HR 1.83, 95% CI 1.46–2.29). Among premenopausal women (18.3% of cases), late first pregnancy combined with high weight gain showed a similar elevated risk (HR 1.70, 95% CI 1.16–2.51), while no significant association was observed for nulliparous women (HR 0.94, 95% CI 0.56–1.58), potentially due to the smaller number of events in the premenopausal nulliparous group (*n* = 42).

A second sensitivity analysis was performed restricting the outcome to oestrogen receptor positive (ER + ve) BCs (*n* = 1414; 83.1% of total cases). Participants with ER‐ve or unknown status cancers were censored at the date of diagnosis. The results were consistent with the primary analysis: women with a late FP and high AWG had a significantly increased risk (HR: 1.79, 95% CI: 1.44–2.21), as did nulliparous women with high AWG (HR: 1.57, 95% CI: 1.25–1.97), confirming the associations are robust within the ER + ve subtype.

### 3.6 Likelihood Ratio Test

A likelihood ratio test confirmed that including a multiplicative interaction term for parity and weight change did not improve the model for BC risk (*p* = 0.69). Consequently, the term was not included in the final model.

## 4 Discussion

### 4.1 Summary of Main Findings

This study has confirmed findings previously reported in the literature—namely (i) early age of first pregnancy is associated with subsequent increased AWG compared with those with late age of first pregnancy [6]; (ii) AWG is associated with increased risk of BC [13]; and (iii) late age of first pregnancy and nulliparity are associated with increased risk of BC [14]. Notably, our results indicate that this risk is not limited to extreme weight gain; statistically significant increases in risk were observed even with modest AWG (5%–15%).

Consistent with the literature, our study confirmed that an early FP protects against postmenopausal BC but is also associated with increased weight gain. In turn, AWG is associated with an increased risk of postmenopausal BC in this cohort, with the strongest independent risk observed in women who gained > 30% of their baseline weight. This study is the first to quantify how the combination of AWG and age of FP can affect a woman's risk of postmenopausal BC. For example, for women who are nulliparous or have a late FP with AWG > 30%, there is a near three‐fold increase in BC risk compared with women with an early FP and either stable or low AWG (< 5%).

However, we did not demonstrate evidence of a statistically significant interaction effect, particularly when viewing weight gain as a continuous variable. Interaction effects are known to require considerably larger sample sizes and event numbers to establish compared to main effect tests [15], so further research is required to determine whether this result reflects an insufficient number of events within this sample. Although the overall number of events was high, the relatively small proportion of participants and events for those with low AWG or a late FP is a limitation of this study.

Although the interaction was not statistically significant, the trend suggested a more pronounced relationship on the additive scale compared to the multiplicative scale. Historically, interactions have often been expressed on the multiplicative scale, although there is evidence that the additive scale may be more appropriate when assessing biological risk [16] and is more useful when determining the public health significance of risk factors [17].

### 4.2 Context of Other Studies

The study adds to the body of literature on weight gain and risk of postmenopausal BC [13]. This updated analysis from the PROCAS study aligns with previous findings with greater event numbers than previously reported [18]; and linkage with the Somerset Cancer Registry, the NWCIS database and the National Breast Screening System. Our results add to these findings by providing evidence that this association exists regardless of age of first pregnancy or parity status. The risk reducing effects of an early pregnancy lead to breast cells becoming less susceptible to cancer development through breast differentiation, reduced hormone responsiveness, changes to the stroma, and reduced progenitor and stem cell activity [19, 20]. However, these permanent structural protections do not appear to mitigate the subsequent tumor‐promoting effects of AWG. So, while an early pregnancy appears to lower the baseline risk of BC, it does not prevent the later tumor‐promoting effects of adiposity.

This lack of interaction differs from the dynamics of other early‐life factors; for example, high body weight in early adulthood has been associated with a reduced impact of later AWG on BC risk, in this [18] and other cohorts [21]. High early life weight is thought to reduce subsequent BC risk through effects on number of pathways including reduced IGF‐1, mammographic density, progesterone and reduced RANK ligand as well as growth factors: bone morphogenetic protein 2 (BMP2) [22, 23, 24, 25].

Our stratified analysis suggests potentially distinct biological mechanisms for these high‐risk groups. For women with a late first pregnancy, the non‐significant trend toward an interaction with high weight gain (RERI: 0.32) may reflect the interplay between adiposity‐related inflammation and the pro‐tumorigenic environment of post‐partum involution, which is known to be more pro‐inflammatory in women with a later first pregnancy [26, 27]. In contrast, nulliparous women exhibited a consistently high risk that appeared independent of weight gain. This likely reflects the lifelong exposure to undifferentiated breast tissue and higher cumulative oestrogen exposure associated with nulliparity [28], which confers a distinct baseline risk that operates independently of AWG.

A small number of previous studies have considered the effect of both BMI & parity on BC risk. One of these, a large cohort study of 58,191 women from Norway, found some evidence of a synergistic effect of a high BMI and nulliparity on BC risk, but only in women over the age of 70 [29]. However, the confidence intervals for this effect were close to 0 (Attributable Risk: 0.21, 95% CI: 0.04–0.39), with confirmation needed from further studies.

While our exposure represents cumulative, general AWG, the perinatal period is an established critical window that contributes to this long‐term weight trajectory. A study in Finland examining the specific impact of gestational weight gain reported that it was similarly associated with an increased risk of postmenopausal BC [30]. Mothers in the upper tertile of pregnancy weight gain (> 15 kg) had a 1.62‐fold (95% CI 1.03–2.53) greater risk compared to mothers who gained the recommended amount of weight (mean: 12.9 kg, range 11–15 kg). Our findings align with the broader concept that weight gained and retained around pregnancy can have lasting implications for BC risk and do not appear to be mitigated by any effects of the pregnancy.

The finding that women with an early FP are more likely to gain weight confirms the results from a US study [6] that found women who have pregnancies later in life were less likely to retain weight during pregnancy. Although we adjusted for age at recruitment to account for the trend of having a FP later, it is possible that other factors such as education level or social deprivation may also be playing a part in this relationship, with further research needed.

### 4.3 Limitations and Strengths

The study has several limitations. First, there was a lack of ethnic diversity, with 92.7% of study participants self‐reporting as white. As the relationship between BMI and BC risk varies by ethnicity [31], the results found may not be generalizable. Second, there were relatively few participants with a late FP or who were nulliparous due to the parity trends at the time participants in this cohort were of childbearing age. This has been identified as a frequent weakness in a number of studies assessing the effect of BC risk on pregnancy age [32]. The small number of participants classed as obese who also had a late FP or were nulliparous was a particular limitation. The continuing trend of women having their FP later in life [33] may mean future studies experience fewer challenges related to this limitation.

We used a DAG to identify confounders, highlighting SES as a critical link between reproductive history and weight gain. To address this, we adjusted for the IMD and lifestyle factors. However, the lack of individual‐level education or income data remains a limitation; while IMD captures area‐level deprivation, residual confounding from individual SES cannot be ruled out. Furthermore, the use of CCA assumes that missing data occurred completely at random. If data missingness was associated with specific participant characteristics (e.g., if women with higher BMIs were less likely to complete all questionnaire items) their exclusion may have introduced selection bias, potentially affecting the generalizability of the findings.

Another potential limitation is that our primary outcome combined all BC subtypes. To mitigate this limitation, we performed a sensitivity analysis restricted to ER+ tumors (83.1% of cases). The results were similar to our primary findings. These observations suggest that our overall findings are driven by the hormone‐receptor‐positive majority.

Strengths of the present study include (first) its prospective design and a contemporary population. Second, we assessed the effect of AWG, which is considered more informative than BMI at a single time point and is able to more accurately capture increases in body fat [23]. The use of self‐reported recall to approximate BMI at age 20 with the PROCAS dataset may be considered a weakness as it could be subject to bias. This is a particular concern as studies have found that participants are likely to over report their height and under report their weight, with this effect increasing with age [34]. However, a recent meta‐analysis of 15 studies that compared recall BMI, height and weight with prospective measures [35] found relatively minor mean pooled differences between the measures, indicating that the use of self‐reported recall weight should be considered methodologically valid.

### 4.4 Clinical Implications

This study highlights the potential additive BC risk, particularly for those with high AWG (> 30%) who have a late first pregnancy or are nulliparous. While our study did not directly assess the impact of weight loss on risk reversal, this combined high‐risk profile identifies a specific group of women who should be prioritized for weight management support and targeted surveillance. Although it could be argued that weight control and health behavior advice is relevant to all women due to the prevention of weight‐related conditions such as diabetes, cardiovascular disease, other cancers and mental wellbeing, the findings could help target preventive lifestyle interventions aimed at BC risk reduction.

Given the long‐term risks highlighted in this study, preventing excessive weight gain during and after pregnancy is a critical public health goal. The perinatal period represents a key opportunity for intervention, and further research into effective weight management strategies for new mothers is essential.

### 4.5 Conclusion and Future Directions

Our study confirms that AWG is an independent, modifiable risk factor for postmenopausal BC across all reproductive histories, reinforcing the importance of weight management advice for all women. While we did not demonstrate a statistical interaction between these factors, the combination of high AWG (> 30%) and late or no pregnancy identifies a specific, high‐risk profile. While general lifestyle interventions are universally beneficial, women in this combined high‐risk group could be prioritized for targeted lifestyle interventions and weight management support in a BC prevention setting.

## Author Contributions

**Lee Malcomson:** investigation, methodology, writing – review and editing, visualization, software, formal analysis, project administration, writing – original draft. **Adam Brentnall:** methodology, formal analysis, supervision, writing – review and editing, conceptualization, investigation. **Andrew G. Renehan:** writing – review and editing, methodology, investigation, validation. **Mary Pegington:** writing – review and editing, methodology, investigation. **Elaine F. Harkness:** data curation, writing – review and editing, methodology, validation. **Jake Southworth:** data curation, writing – review and editing, project administration, resources. **D. Gareth Evans:** writing – review and editing, data curation, resources. **Michelle Harvie:** supervision, data curation, conceptualization, investigation, funding acquisition, writing – original draft, writing – review and editing, methodology.

## Funding

This research was funded by the NIHR Manchester Biomedical Research Centre (NIHR203308). The views expressed are those of the author(s) and not necessarily those of the NIHR or the Department of Health and Social Care.

## Ethics Statement

The PROCAS study received ethical approval from the Central Manchester Research Ethics Committee (reference: 09/H1008/81). All participants provided written informed consent for their data to be used for research. The study is registered with the ISRCTN (reference: ISRCTN91372184).

## Conflicts of Interest

Adam Brentnall receives royalty payments through Cancer Research UK for the commercial use of the IBIS (Tyrer‐Cuzick) breast cancer risk algorithm. The other authors declare no conflicts of interest.

## Supporting information


**Table S1:** Hazard Ratios for the association between continuous adult weight gain and breast cancer risk.
**Table S2:** Sensitivity analysis of combined risk groups stratified by menopausal status at diagnosis.
**Table S3:** Sensitivity analysis of combined risk groups adjusted for age at recruitment only.
**Figure S1:** Directed Acyclic Graph (DAG) for the assumed causal relationships between age at first pregnancy, adult weight gain, and breast cancer risk.
**Figure S2:** Breast cancer diagnosis by parity status.
**Figure S3:** Breast cancer diagnosis by weight change categories.

## Data Availability

All source code is publicly available on GitHub via: https://github.com/LeeMalc/PROCAS/blob/8764008879ee6c1756d2dbfb50e3f839dc043392/parity_weight_gain. Deidentified data is available from the PROCAS Research Group after approval from the University of Manchester. Further information is available from the corresponding author upon reasonable request.

## References

[1] Cancer Research UK , “Breast Cancer Incidence (Invasive) Statistics, 2020”, https://www.cancerresearchuk.org/health‐professional/cancer‐statistics/statistics‐by‐cancer‐type/breast‐cancer/incidence‐invasive#ref‐2.

[2] World Cancer Research Fund/American Institute for Cancer Research . Diet, Nutrition, Physical Activity and Cancer: A Global Perspective (World Cancer Research Fund International, 2018).

[3] M. Lambertini , L. Santoro , L. Del Mastro , et al., “Reproductive Behaviors and Risk of Developing Breast Cancer According to Tumor Subtype: A Systematic Review and Meta‐Analysis of Epidemiological Studies,” Cancer Treatment Reviews 49 (2016): 65–76. 27529149 10.1016/j.ctrv.2016.07.006

[4] M. Pegington , D. P. French , and M. N. Harvie , “Why Young Women Gain Weight: A Narrative Review of Influencing Factors and Possible Solutions,” Obesity Reviews 21, no. 5 (2020): e13002. 32011105 10.1111/obr.13002

[5] J. L. Hollis , S. R. Crozier , H. M. Inskip , et al., “Modifiable Risk Factors of Maternal Postpartum Weight Retention: An Analysis of Their Combined Impact and Potential Opportunities for Prevention,” International Journal of Obesity 41, no. 7 (2017): 1091–1098. 28337028 10.1038/ijo.2017.78 PMC5500180

[6] L. K. Endres , H. Straub , C. McKinney , et al., “Postpartum Weight Retention Risk Factors and Relationship to Obesity at 1 Year,” Obstetrics & Gynecology 125, no. 1 (2015): 144–152. 25560116 10.1097/AOG.0000000000000565 PMC4286308

[7] NHS Digital , ed., “Health Survey for England, 2021: Data Tables,” in Health Survey for England, 2021. London: NHS Digital 2022.

[8] ONS U , “Birth statistics: England and Wales, 2023”, https://www.ons.gov.uk/peoplepopulationandcommunity/birthsdeathsandmarriages/livebirths/bulletins/birthsummarytablesenglandandwales/2022.

[9] D. G. Evans , S. Astley , P. Stavrinos , et al., Programme Grants for Applied Research. Improvement in Risk Prediction, Early Detection and Prevention of Breast Cancer in the NHS Breast Screening Programme and Family History Clinics: A Dual Cohort Study (NIHR Journals Library, 2016). 27559559

[10] Cancer CGoHFiB , “Menarche, Menopause, and Breast Cancer Risk: Individual Participant Meta‐Analysis, Including 118 964 Women With Breast Cancer From 117 Epidemiological Studies,” Lancet Oncology 13, no. 11 (2012): 1141–1151. 23084519 10.1016/S1470-2045(12)70425-4 PMC3488186

[11] Cancer CGoHFiB , “Type and Timing of Menopausal Hormone Therapy and Breast Cancer Risk: Individual Participant Meta‐Analysis of the Worldwide Epidemiological Evidence,” Lancet 394, no. 10204 (2019): 1159–1168. 31474332 10.1016/S0140-6736(19)31709-X PMC6891893

[12] E. von Elm , D. G. Altman , M. Egger , S. J. Pocock , P. C. Gøtzsche , and J. P. Vandenbroucke , “The Strengthening the Reporting of Observational Studies in Epidemiology (STROBE) Statement: Guidelines for Reporting Observational Studies,” Lancet 370, no. 9596 (2007): 1453–1457. 18064739 10.1016/S0140-6736(07)61602-X

[13] N. Keum , D. C. Greenwood , D. H. Lee , et al., “Adult Weight Gain and Adiposity‐Related Cancers: A Dose‐Response Meta‐Analysis of Prospective Observational Studies,” Journal of the National Cancer Institute 107, no. 2 (2015): djv088. 25757865 10.1093/jnci/djv088

[14] G. Albrektsen , I. Heuch , S. Hansen , and G. Kvåle , “Breast Cancer Risk by Age at Birth, Time Since Birth and Time Intervals Between Births: Exploring Interaction Effects,” British Journal of Cancer 92, no. 1 (2005): 167–175. 15597097 10.1038/sj.bjc.6602302 PMC2361726

[15] B. Peterson and S. L. George , “Sample Size Requirements and Length of Study for Testing Interaction in a 2 × k Factorial Design When Time‐To‐Failure Is the Outcome [Corrected],” Controlled Clinical Trials 14, no. 6 (1993): 511–522. 8119066 10.1016/0197-2456(93)90031-8

[16] M. J. Knol , T. J. VanderWeele , R. H. Groenwold , O. H. Klungel , M. M. Rovers , and D. E. Grobbee , “Estimating Measures of Interaction on an Additive Scale for Preventive Exposures,” European Journal of Epidemiology 26, no. 6 (2011): 433–438. 21344323 10.1007/s10654-011-9554-9 PMC3115067

[17] T. J. VanderWeele and M. J. Knol , “A Tutorial on Interaction,” Epidemiological Methods 3, no. 1 (2014): 33–72.

[18] A. G. Renehan , M. Pegington , M. N. Harvie , et al., “Young Adulthood Body Mass Index, Adult Weight Gain and Breast Cancer Risk: The PROCAS Study (United Kingdom),” British Journal of Cancer 122, no. 10 (2020): 1552–1561. 32203222 10.1038/s41416-020-0807-9 PMC7217761

[19] I. H. Russo and J. Russo , “Pregnancy‐Induced Changes in Breast Cancer Risk,” Journal of Mammary Gland Biology and Neoplasia 16, no. 3 (2011): 221–233. 21805333 10.1007/s10911-011-9228-y

[20] F. Meier‐Abt , M. Bentires‐Alj , and C. Rochlitz , “Breast Cancer Prevention: Lessons To Be Learned From Mechanisms of Early Pregnancy‐Mediated Breast Cancer Protection,” Cancer Research 75, no. 5 (2015): 803–807. 25660950 10.1158/0008-5472.CAN-14-2717

[21] A. H. Eliassen , G. A. Colditz , B. Rosner , W. C. Willett , and S. E. Hankinson , “Adult Weight Change and Risk of Postmenopausal Breast Cancer,” JAMA 296, no. 2 (2006): 193–201. 16835425 10.1001/jama.296.2.193

[22] Y. Han , G. A. Colditz , and A. T. Toriola , “Changes in Adiposity Over the Life Course and Gene Expression in Postmenopausal Women,” Cancer Medicine 11, no. 13 (2022): 2699–2710. 35304837 10.1002/cam4.4649 PMC9249983

[23] E. M. Poole , S. S. Tworoger , S. E. Hankinson , E. S. Schernhammer , M. N. Pollak , and H. J. Baer , “Body Size in Early Life and Adult Levels of Insulin‐Like Growth Factor 1 and Insulin‐Like Growth Factor Binding Protein 3,” American Journal of Epidemiology 174, no. 6 (2011): 642–651. 21828371 10.1093/aje/kwr123 PMC3166705

[24] K. A. Bertrand , H. J. Baer , E. J. Orav , et al., “Body Fatness During Childhood and Adolescence and Breast Density in Young Women: A Prospective Analysis,” Breast Cancer Research 17, no. 1 (2015): 95. 26174168 10.1186/s13058-015-0601-4 PMC4502611

[25] M. Dowsett and E. Folkerd , “Reduced Progesterone Levels Explain the Reduced Risk of Breast Cancer in Obese Premenopausal Women: A New Hypothesis,” Breast Cancer Research and Treatment 149, no. 1 (2015): 1–4. 25414027 10.1007/s10549-014-3211-4

[26] P. Schedin , “Pregnancy‐Associated Breast Cancer and Metastasis,” Nature Reviews. Cancer 6, no. 4 (2006): 281–291. 16557280 10.1038/nrc1839

[27] T. R. Lyons , J. O'Brien , V. F. Borges , et al., “Postpartum Mammary Gland Involution Drives Progression of Ductal Carcinoma in Situ Through Collagen and COX‐2,” Nature Medicine 17, no. 9 (2011): 1109–1115. 10.1038/nm.2416 PMC3888478 21822285

[28] K. Britt , A. Ashworth , and M. Smalley , “Pregnancy and the Risk of Breast Cancer,” Endocrine‐Related Cancer 14, no. 4 (2007): 907–933. 18045947 10.1677/ERC-07-0137

[29] S. Opdahl , M. D. Alsaker , I. Janszky , P. R. Romundstad , and L. J. Vatten , “Joint Effects of Nulliparity and Other Breast Cancer Risk Factors,” British Journal of Cancer 105, no. 5 (2011): 731–736. 21811252 10.1038/bjc.2011.286 PMC3188938

[30] T. I. Kinnunen , R. Luoto , M. Gissler , E. Hemminki , and L. Hilakivi‐Clarke , “Pregnancy Weight Gain and Breast Cancer Risk,” BMC Women's Health 4, no. 1 (2004): 7. 15498103 10.1186/1472-6874-4-7 PMC535935

[31] E. V. Bandera , G. Maskarinec , I. Romieu , and E. M. John , “Racial and Ethnic Disparities in the Impact of Obesity on Breast Cancer Risk and Survival: A Global Perspective,” Advances in Nutrition 6, no. 6 (2015): 803–819. 26567202 10.3945/an.115.009647 PMC4642425

[32] K. Rojas and A. Stuckey , “Breast Cancer Epidemiology and Risk Factors,” Clinical Obstetrics and Gynecology 59, no. 4 (2016): 651–672. 27681694 10.1097/GRF.0000000000000239

[33] A. E. P. Heazell , L. Newman , S. C. Lean , and R. L. Jones , “Pregnancy Outcome in Mothers Over the Age of 35,” Current Opinion in Obstetrics & Gynecology 30, no. 6 (2018): 337–343. 30239372 10.1097/GCO.0000000000000494

[34] R. M. Merrill and J. S. Richardson , “Validity of Self‐Reported Height, Weight, and Body Mass Index: Findings From the National Health and Nutrition Examination Survey, 2001‐2006,” Preventing Chronic Disease 6, no. 4 (2009): A121. 19754997 PMC2774635

[35] V. De Rubeis , S. Bayat , L. E. Griffith , B. T. Smith , and L. N. Anderson , “Validity of Self‐Reported Recall of Anthropometric Measures in Early Life: A Systematic Review and Meta‐Analysis,” Obesity Reviews 20, no. 10 (2019): 1426–1440. 31184422 10.1111/obr.12881

