# Supplementary material for: Age at First Pregnancy, Adult Weight Gain and Postmenopausal Breast Cancer Risk: The PROCAS Study (United Kingdom)
Source: Int J Cancer. 2026 Apr 12;159(5):1228–39. doi: 10.1002/ijc.70489 (PMC13340970; doi:10.1002/ijc.70489)
Supplement: Supplementary file 1 — Table S1: Hazard Ratios for the association between continuous adult weight gain and breast cancer risk. Table S2: Sensitivity analysis of combined risk groups stratified by menopausal status at diagnosis. Table S3: Sensitivity analysis of combined risk groups adjusted for age at recruitment only. Figure S1: Directed Acyclic Graph (DAG) for the assumed causal relationships between age at first pregnancy, adult weight gain, and breast cancer risk. Figure S2: Breast cancer diagnosis by parity status. Figure S3: Breast cancer diagnosis by weight change categories. [file IJC-159-1228-s001.pdf]

SUPPLEMENTARY MATERIALS

Age at first pregnancy, adult weight gain and post-menopausal breast cancer risk: The PROCAS Study (United Kingdom)

Lee Malcomson, Adam Brentnall, Andrew G Renehan, Mary Pegington, Elaine F Harkness
Jake Southworth, D Gareth Evans, Michelle Harvie

Contents

Table S1: Hazard Ratios for the association between continuous adult weight gain and breast cancer risk 2

Table S2: Sensitivity analysis of combined risk groups stratified by menopausal status at diagnosis. 3

Table S3: Sensitivity analysis of combined risk groups adjusted for age at recruitment only 4

Figure S1: Directed Acyclic Graph (DAG) for the assumed causal relationships between age at first pregnancy, adult weight gain, and breast cancer risk 5

Figure S2 - Breast cancer diagnosis by parity status 6

Figure S3 - Breast cancer diagnosis by weight change categories 7

STROBE Statement - checklist of items that should be included in reports of observational studies 8

Table S1: Hazard Ratios for the association between continuous adult weight gain and breast cancer risk

	HR (95% CI)	P-value
**Model A: Relative Weight Change (%)**		
**Relative adult weight change (per 10%)**	1.07 (1.04 – 1.10)	< 0.001
**Parity: Late First Pregnancy (vs Early)**	1.19 (0.95 – 1.49)	0.13
**Parity: Nulliparous (vs Early)**	1.25 (1.00 – 1.56)	0.05
**Interaction: Weight Change × Late FP**	1.01 (0.95 – 1.08)	0.68
**Interaction: Weight Change × Nulliparous**	1.00 (0.94 – 1.07)	0.96
**Age at First Pregnancy (per year)***	1.02 (1.00 – 1.04)	0.04
**Model B: Absolute Weight Change (kg)**		
**Absolute weight change (per kg)**	1.01 (1.01 – 1.02)	< 0.001
**Parity: Late First Pregnancy (vs Early)**	1.17 (0.94 – 1.47)	0.16
**Parity: Nulliparous (vs Early)**	1.24 (0.99 – 1.54)	0.06
**Interaction: Absolute Weight × Late FP**	1.00 (0.99 – 1.02)	0.57
**Interaction: Absolute Weight × Nulliparous**	1.00 (0.99 – 1.01)	0.87
**Age at First Pregnancy (per year)***	1.02 (1.00 – 1.03)	0.06

***Amongst parous participants**

Adjusted for: recruitment age + HRT use * BMI at 20 + Menopausal status + alcohol + exercise + menarche age + ethnicity + height + family history + Index of Multiple Deprivation

Table S2: Sensitivity analysis of combined risk groups stratified by menopausal status at diagnosis.

Risk Group (vs Early FP & Low adult)	Post-Menopausal HR (95% CI)	Pre-Menopausal HR (95% CI)
**Late FP & Low adult weight gain (< 15%)**	1.68 (1.33–2.11)	1.70 (1.16–2.51)
**Late FP & High adult weight gain (>= 15%)**	1.83 (1.46–2.29)	0.94 (0.56–1.58)

Post-menopausal defined as peri/post-menopausal at recruitment or diagnosis age >55. Pre-menopausal defined as pre-menopausal at recruitment and diagnosis age <55.

Models adjusted for recruitment age + HRT use * BMI at 20 + Menopausal status + alcohol + exercise + menarche age + ethnicity + height + family history + Index of Multiple Deprivation

Table S3: Sensitivity analysis of combined risk groups adjusted for age at recruitment only

Risk Group (vs Early FP & Low AWG)	Age-Adjusted HR (95% CI)	P-value
**Early First Pregnancy**		
**Low adult weight gain (< 15%)**	1.00 [Reference]	-
**High adult weight gain (≥ 15%)**	1.32 (1.16 – 1.50)	< 0.001
**Late First Pregnancy (≥ 30 years)**		
**Low adult weight gain (< 15%)**	1.08 (0.84 – 1.38)	0.544
**High adult weight gain (≥ 15%)**	1.81 (1.52 – 2.17)	< 0.001
**Nulliparous**		
**Low adult weight gain (< 15%)**	1.29 (1.02 – 1.63)	0.037
**High adult weight gain (≥ 15%)**	1.65 (1.36 – 2.00)	< 0.001

Model adjusted for recruitment age only

Figure S1: Directed Acyclic Graph (DAG) for the assumed causal relationships between age at first pregnancy, adult weight gain, and breast cancer risk.

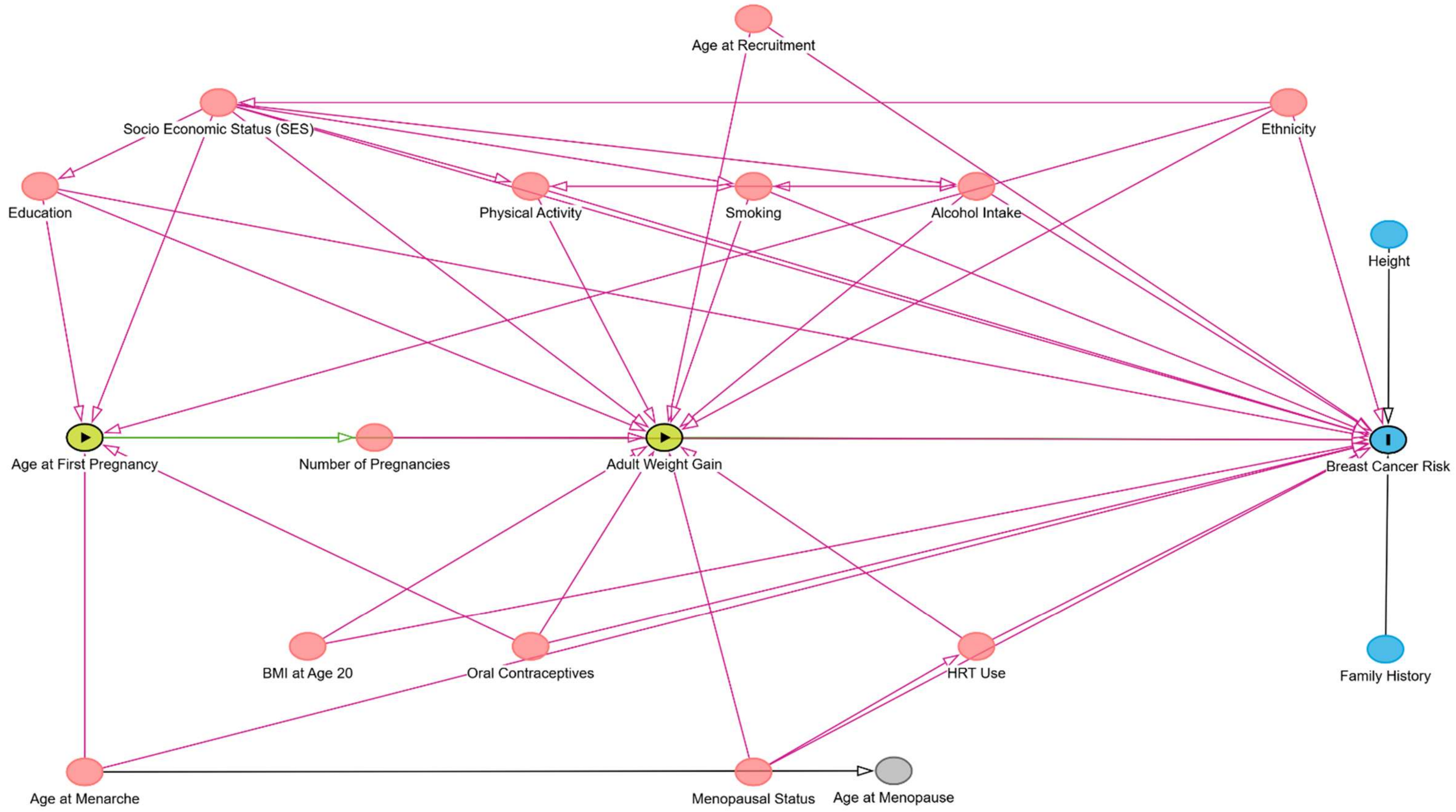

The DAG was used to identify the minimal sufficient adjustment set for the multivariable analysis. Arrows represent assumed causal pathways based on a priori biological and epidemiological knowledge. Nodes in pink represent variables identified by the causal structure as potential confounders or key ancestors. The majority of these were included in the final adjustment set (e.g., Socioeconomic Status/IMD, Alcohol Intake, Physical Activity). “Number of Pregnancies" was identified as a mediator on the causal pathway between Age at First Pregnancy and Adult Weight Gain and was therefore excluded from the primary model to avoid over-adjustment bias. "Education" and "Smoking" were identified as potential confounders but were unavailable in the dataset; IMD was used as a proxy for socioeconomic confounding.

Figure S2 - Breast cancer diagnosis by parity status

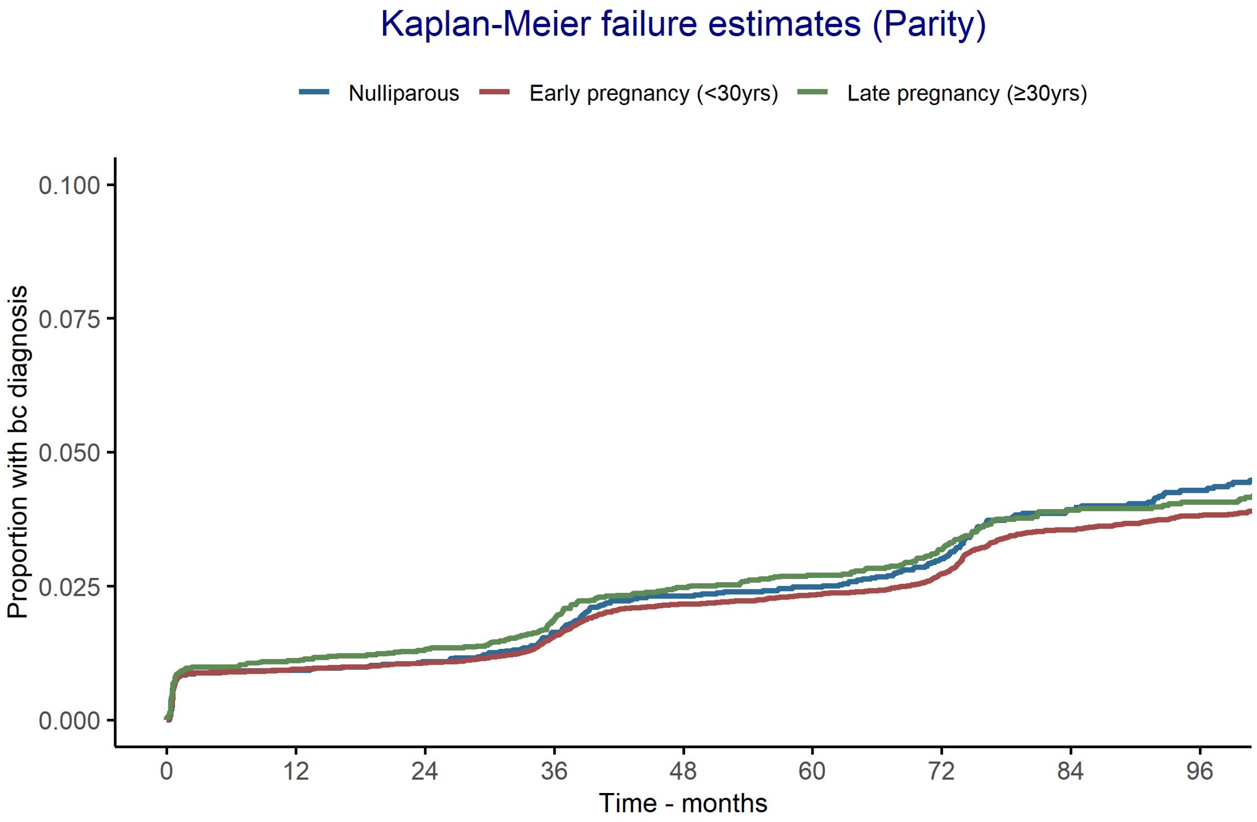

Number at risk

Nulliparous	6071	5438	5403	5046	4637	4571	3975	2802	2637
Early pregnancy (<30yrs)	35442	31242	31045	28983	26257	25854	22510	15821	14911
Late pregnancy (≥30yrs)	6711	6134	6115	5786	5442	5377	4707	3273	3139

Figure S3 - Breast cancer diagnosis by weight change categories

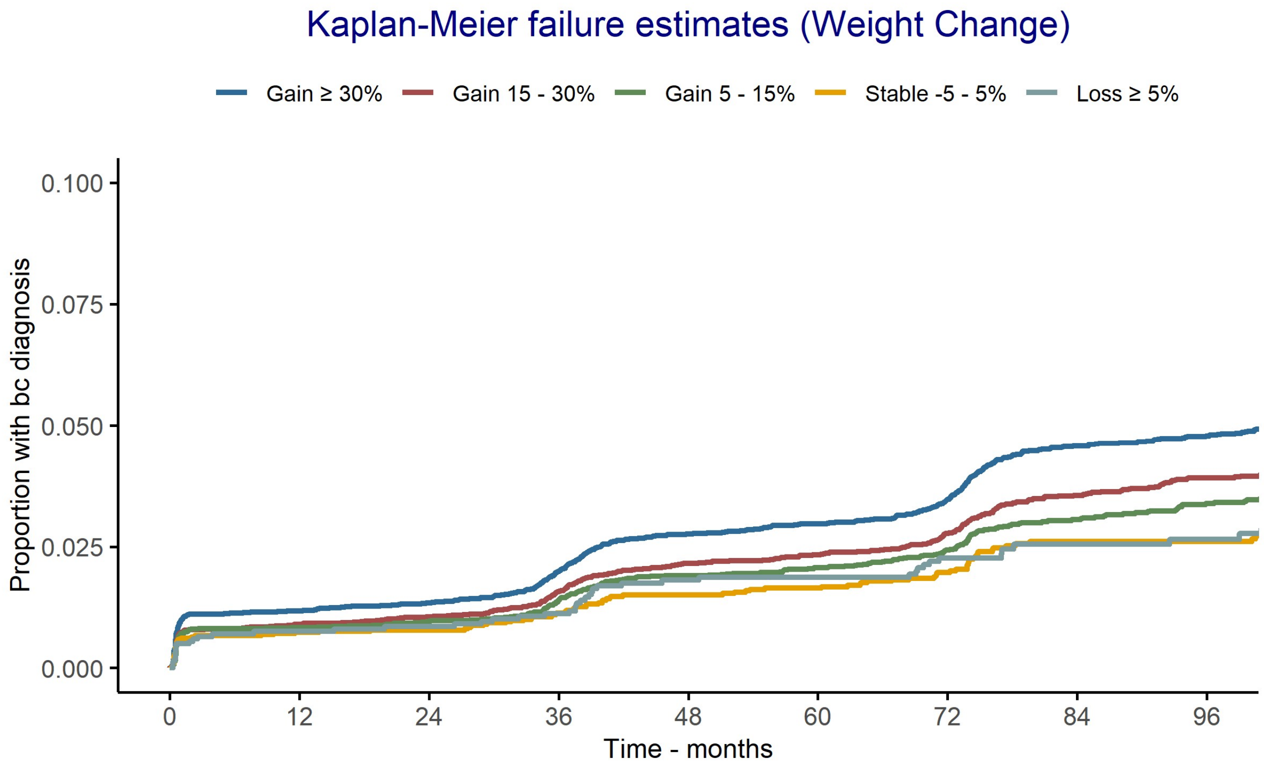

Number at risk

16155	14255	14169	13245	12034	11847	10227	7123	6683
14737	13154	13069	12233	11159	11001	9629	6880	6530
10451	9321	9274	8697	7965	7861	6889	4832	4602
4652	4134	4117	3844	3556	3502	3081	2097	1982
2229	1950	1934	1796	1622	1591	1366	964	890

STROBE Statement - checklist of items that should be included in reports of observational studies

	Item No.	Recommendation	Page No.	Relevant text from manuscript
**Title and abstract**	1	(*a*) Indicate the study's design with a commonly used term in the title or the abstract	1	"We analysed prospective data from 48,417 women in the PROCAS cohort..."
		(*b*) Provide in the abstract an informative and balanced summary of what was done and what was found	1	"Background: Adult weight gain (AWG) increases postmenopausal breast cancer (BC) risk, whereas an early first pregnancy (FP) is protective... Methods: We analysed prospective data from 48,417 women in the PROCAS cohort... Results: ...Compared to women with an early FP (<30 years) and minimal AWG (<5%), those with high AWG (>30%) and a late FP (≥30 years) or nulliparous status had a substantially higher risk... However, there was no statistical evidence of an interaction between these two factors... Conclusions: Maintaining a stable adult weight and an earlier FP are independently associated with lower BC risk..."
**Introduction**				
Background/rationale	2	Explain the scientific background and rationale for the investigation being reported	1-2	"Two established risk factors for post-menopausal BC are adult weight gain (AWG) (2) and a first pregnancy (FP) at a relatively late age (>30 years) or nulliparity (3)." "Given these trends and with UK female BC diagnoses at their highest recorded rate (1), there is a need to investigate how these factors interact on BC risk to better inform and target weight control advice..."
Objectives	3	State specific objectives, including any prespecified hypotheses	2	"This study aimed to determine whether the association between AWG and BC risk is modified by age at FP, specifically whether early FP attenuates the adverse effect of AWG."
**Methods**				
Study design	4	Present key elements of study design early in the paper	2	"The Predicting Risk of Breast Cancer at Screening (PROCAS) cohort study... recruited 57,902 women aged 46 - 84 years from the Greater Manchester Breast Screening Programme between October 2009 and June 2015."
Setting	5	Describe the setting, locations, and relevant dates, including periods of recruitment,	2-3	Recruitment: "...from the Greater Manchester Breast Screening Programme between October 2009 and June 2015." Follow-up: "The study followed participants from their PROCAS enrolment date... to a final data query for any cancer diagnoses performed in January 2022."

		exposure, follow-up, and data collection		
Participants	6	(*a*) *Cohort study*—Give the eligibility criteria, and the sources and methods of selection of participants. Describe methods of follow-up<br><br>*Case-control study*—Give the eligibility criteria, and the sources and methods of case ascertainment and control selection. Give the rationale for the choice of cases and controls<br><br>*Cross-sectional study*—Give the eligibility criteria, and the sources and methods of selection of participants	2-3	Eligibility criteria: "...women aged 46 - 84 years from the Greater Manchester Breast Screening Programme..." Follow-up: "Cancer events were identified through the Somerset Cancer Registry and the North-West Cancer Intelligence Service (NWCIS)." "For event-free participants, the date of their most recent mammogram, obtained from the National Breast Screening System (NBSS), was used as the censoring date for follow-up."
		(*b*) *Cohort study*—For matched studies, give matching criteria and number of exposed and unexposed<br><br>*Case-control study*—For matched studies, give matching criteria and the number of controls per case	N/A	
Variables	7	Clearly define all outcomes, exposures, predictors, potential confounders, and effect	2-3	Outcome: "The primary outcome was the diagnosis of a new BC, classed as either an invasive or ductal carcinoma in situ (DCIS), diagnosed after the study entry date." Exposures: "Relative adult weight change (%)" and "early FP < 30 years or a late FP ≥ 30 years / nulliparous".

		modifiers. Give diagnostic criteria, if applicable		Confounders: "...recruitment age, BMI at study enrolment, current HRT use...and the interaction of BMI at enrolment and HRT use."
Data sources/ measurement	8*	For each variable of interest, give sources of data and details of methods of assessment (measurement). Describe comparability of assessment methods if there is more than one group	*2-3*	"Each consented participant completed a two-page questionnaire which included self-reported information about BC risk factors." Weight change was "determined from self-reported weights recalled by the participant at age 20, and their self-reported weight at study entry...".
Bias	9	Describe any efforts to address potential sources of bias	6	" However, the lack of individual-level education or income data remains a limitation; while IMD captures area-level deprivation, residual confounding from individual SES cannot be ruled out.." "The use of self-reported recall to approximate BMI at age 20… may be considered a weakness as it could be subject to bias."
Study size	10	Explain how the study size was arrived at	Fig 1	Study flow chart
Quantitati ve variables	11	Explain how quantitative variables were handled in the analyses. If applicable, describe which groupings were chosen and why	2-3	Adult Weight Gain (AWG) was analysed as a categorical variable (e.g., "above and below 15%") and as a continuous measure. Age at first pregnancy was categorised ("early FP < 30 years or a late FP ≥ 30 years / nulliparous"), with the threshold of 30 years supported by a meta-analysis.
Statistical methods	12	(*a*) Describe all statistical methods, including those used to control for confounding	3	"For the primary analysis, Cox Proportional Hazard modelling was used to estimate mean hazard ratios (HR) with 95% confidence intervals (13)." The model was adjusted for covariates including recruitment age and HRT use.
		(*b*) Describe any methods used to examine subgroups and interactions	3	"A potential interaction between high AWG (>15%) and a late FP or nulliparous status on breast cancer risk was assessed on both multiplicative and additive scales." Methods included adding a product term to the model and calculating the RERI, AP, and Synergy Index.
		(*c*) Explain how missing data were addressed	2	"We undertook a Complete Case Analysis (CCA), including 48,417 participants (Figure 1) who provided information on pregnancy status and AWG."

		(*d*) *Cohort study*—If applicable, explain how loss to follow-up was addressed<br><br>*Case-control study*—If applicable, explain how matching of cases and controls was addressed<br><br>*Cross-sectional study*—If applicable, describe analytical methods taking account of sampling strategy	3	"For event-free participants, the date of their most recent mammogram, obtained from the National Breast Screening System (NBSS), was used as the censoring date for follow-up."
		(e) Describe any sensitivity analyses	5	The analysis was repeated "with relative weight change (%) replaced by absolute weight change (kg), which also produced a non-significant result for the interaction term..."
**Results**				
Participan ts	13*	(a) Report numbers of individuals at each stage of study—eg numbers potentially eligible, examined for eligibility, confirmed eligible, included in the study, completing follow-up, and analysed	Fig 1	Figure 1 provides a complete flow diagram
		(b) Give reasons for non-participation at each stage	2 & Fig 1	Reasons for exclusion included withdrawn consent (4) , previous breast cancer (905) , missing data (268, 3,834, 4,372) , and outlier BMI recordings (102).
		(c) Consider use of a flow diagram	Fig 1	Flow diagram
Descriptiv e data	14*	(a) Give characteristics of study participants (eg demographic, clinical, social) and information	4 & Table 1	"The baseline characteristics are shown in Table 1 by parity status for the 48,417 participants included in the analysis."

		on exposures and potential confounders		
		(b) Indicate number of participants with missing data for each variable of interest	Table 1	Table 1 reports the number of participants with missing data for variables like "Ethnicity" and "Alcohol Use".
		(c) *Cohort study*—Summarise follow-up time (eg, average and total amount)	4	"After a median follow-up of 6.4 years..."
Outcome data	15*	*Cohort study*—Report numbers of outcome events or summary measures over time	*4 and table 3*	*"...there were 1,702 new breast cancer diagnoses (1,605 invasive carcinoma and 97 DCIS)." Table 3 also reports the number of breast cancer events (BC) and total participants (N) within each exposure category.*
		Case-control study—Report numbers in each exposure category, or summary measures of exposure		
		Cross-sectional study—Report numbers of outcome events or summary measures		
Main results	16	(*a*) Give unadjusted estimates and, if applicable, confounder-adjusted estimates and their precision (eg, 95% confidence interval). Make clear which confounders were adjusted for and why they were included	Table 2 & 3	Confounder-adjusted Hazard Ratios (HRs) and 95% Confidence Intervals (CIs) are provided in Table 2 and Table 3. The adjustment variables are listed in the table footnotes.
		(*b*) Report category boundaries when continuous variables were categorized	Table 2 & 3	first pregnancy: "< 30yrs" vs "≥ 30yrs". Adult weight gain categories are defined in Table 3 (e.g., "Stable & Loss: ≤5%").

		(*c*) If relevant, consider translating estimates of relative risk into absolute risk for a meaningful time period	N/A	
Other analyses	17	Report other analyses done—eg analyses of subgroups and interactions, and sensitivity analyses	4-5	An interaction analysis was conducted on both multiplicative and additive scales (Table 2). The analysis was repeated with weight change as a continuous variable. A likelihood ratio test was also performed.
**Discussion**				
Key results	18	Summarise key results with reference to study objectives	5	"This study has confirmed findings previously reported… – namely (i) early age of FP is associated with subsequent increased AWG… (ii) AWG is associated with increased risk of BC… and (iii) late age of FP and nulliparity are associated with increased risk of BC." "However, we did not demonstrate evidence of a statistically significant interaction effect…"
Limitation s	19	Discuss limitations of the study, taking into account sources of potential bias or imprecision. Discuss both direction and magnitude of any potential bias	6	Limitations discussed: lack of ethnic diversity, relatively small numbers in some subgroups (e.g., late FP), lack of data on potential confounders, and potential for bias from self-reported recall of weight.
Interpretat ion	20	Give a cautious overall interpretation of results considering objectives, limitations, multiplicity of analyses, results from similar studies, and other relevant evidence	9	"However, we did not demonstrate evidence of a statistically significant interaction effect, particularly when viewing weight gain as a continuous variable. Interaction effects are known to require considerably larger sample size and event numbers to establish compared to main effect tests (15), so further research is required to determine whether this result reflects an insufficient number of events within this sample. Although the overall number of events was high, the relatively small proportion of participants and events for those with low AWG or a late FP is a limitation of this study.."
Generalis ability	21	Discuss the generalisability (external validity) of the study results	6	"First, there was a lack of ethnic diversity, with 92.7% of study participants self-reporting as white. As the relationship between BMI and BC risk varies by ethnicity (30), the results found may not be generalisable."

**Other information**				
Funding	22	Give the source of funding and the role of the funders for the present study and, if applicable, for the original study on which the present article is based	7	"This research was funded by the NIHR Manchester Biomedical Research Centre (NIHR203308). The views expressed are those of the author(s) and not necessarily those of the NIHR or the Department of Health and Social Care."
